# The Effect of Pro-Inflammatory Conditioning and/or High Glucose on Telomere Shortening of Aging Fibroblasts

**DOI:** 10.1371/journal.pone.0073756

**Published:** 2013-09-23

**Authors:** Klelia D. Salpea, Cecilia G. Maubaret, Annegret Kathagen, Gie Ken-Dror, Derek W. Gilroy, Steve E. Humphries

**Affiliations:** 1 Centre for Cardiovascular Genetics, Division of Medicine, University College London, London, United Kingdom; 2 Centre for Clinical Pharmacology and Therapeutics, Division of Medicine, University College London, London, United Kingdom; Universtiy of Maryland Schoool of Medicine, United States of America

## Abstract

Cardiovascular disease and diabetes have been linked to shorter telomeres, but it is not yet clear which risk factors contribute to shorter telomeres in patients. Our aim was to examine whether pro-inflammatory conditioning, in combination or not with high glucose, result in a higher rate of telomere shortening during *in vitro* cellular ageing. Human fibroblasts from four donors were cultured for 90 days in: 1) medium lacking ascorbic acid only, 2) 10 mM buthionine sulphoximine (BSO) (pro-oxidant), 3) 25 mM D-glucose, 4) 1 ng/ml IL1B and 5) 25 mM D-glucose+1 ng/ml IL1B. Telomere length was measured with qPCR and intracellular reactive oxygen species (ROS) content and cell death with flow cytometry. Cultures treated with high glucose and BSO displayed a significantly lower growth rate, and cultures treated with IL1B showed a trend towards a higher growth rate, compared to the control [Glucose:0.14 PD/day, p<0.001, BSO: 0.11 PD/day, p = 0.006 and IL1B: 0.19 PD/day, p = 0.093 vs. Control:0.16 PD/day]. Telomere shortening with time was significantly accelerated in cultures treated with IL1B compared to the control [IL1B:−0.8%/day (95%CI:−1.1, −0.5) vs. Control:−0.6%/day (95%CI:−0.8, −0.3), p = 0.012]. The hastening of telomere shortening by IL1B was only in part attenuated after adjustment for the number of cell divisions [IL1B:−4.1%/PD (95%CI:−5.7, −2.4) vs. Control:−2.5%/PD (95%CI:−4.4, −0.7), p = 0.067]. The intracellular ROS content displayed 69% increase (p = 0.033) in BSO compared to the control. In aging fibroblasts, pro-inflammatory conditioning aggravates the shortening of telomeres, an effect which was only in part driven by increased cell turnover. High glucose alone did not result in greater production of ROS or telomere shortening.

## Introduction

Tissue aging and cellular senescence play a major role in the pathology of cardiovascular disease (CVD) [1] and type 2 diabetes (T2D) [2]. A widely accepted mechanism of replicative senescence in human somatic cells, which is linked to aging *in vivo*, is telomere shortening [3] [4]. In recent years, a large number of studies have provided evidence for an association of short telomere length with CVD and T2D [5], yet, it is still unclear how these short telomeres arise in those predisposed to, or already with, CVD or T2D.

Inheritance may in part determine the length of telomeres, but non-heritable factors also have a considerable impact on telomere length dynamics during life [6], [7]. Previous association studies have shown that telomere length reflects the lifelong accumulating burden of increased oxidative stress and inflammation [8]. However, the mechanisms leading to short telomeres at the cellular level, the factors determining the rate of shortening and how these are related to the development of vascular diseases or type 2 diabetes remain to be elucidated.

Our aim was to shed light on these mechanisms and thus, we decided to carry out *in vitro* experiments investigating what CVD/T2D risk factors cause telomere shortening during aging at the cellular level. Our hypothesis was that factors having an effect on telomeres may be arising from metabolic disorders, linked to increased nutrient concentration, or chronic inflammation.

On the one hand, increased nutrient availability (e.g. glucose) potentially leads to a higher rate of oxidative phosphorylation in the mitochondria and/or a consequent higher production of reactive oxygen species (ROS) as byproducts. Also, an increased catabolism of these nutrients is likely to lead to an increase of the NADPH-to-NADP ratio and a consequent enhancement of NADPH oxidase-dependent production of superoxide. The high levels of ROS are known to cause oxidative DNA damage, and therefore to result in greater loss of telomeric sequences during replication as shown *in vitro* [9], [10], [11]. Nevertheless, the data on whether hyperglycaemia elicits an increase in intracellular ROS production are conflicting; Other studies provide evidence supporting that glucose induces an increase in ROS generation [12], [13], [14], while other studies argue that this phenomenon occurs under all circumstances [15], [16].

On the other hand, pro-inflammatory cytokines may lead to a higher rate of telomeric loss through the stimulation of a sustained cell turnover. Inflammatory cytokines, such as interleukin 1 beta (IL1B), have a key regulatory role in the atherosclerotic process [17] and are also implicated in the failure of β-cells during diabetes development [18].

Therefore, the present study sought to examine whether high concentration of a basic mitochondrial substrate, such as glucose, or pro-inflammatory conditioning with IL1B, cause greater telomere erosion in a well described *in vitro* model of cellular ageing (i.e. telomerase is not expressed), the human skin fibroblast [19]. More specifically, our purpose was to examine the effect of each of the above stress *stimuli* separately and in combination, since the co-existence of an inflammatory state with high glucose levels is the most common situation in patients with or predisposed to CVD and T2D. The investigation of this treatment combination was also interesting due to the study of Busik et al. [16], which showed in endothelial cell cultures that high glucose alone may not result in augmentation of ROS production, whereas the pro-inflammatory cytokine IL1B may trigger higher glucose consumption by the respiratory chain, and thus to an enhancement of ROS production.

For the *in vitro* modelling of oxidative stress, an inhibitor of γ-glutamyl cysteine synthase, buthionine sulphoximine (BSO), was employed [20]; γ-glutamyl cysteine synthase is a key enzyme of the glutathione redox-cycle, which is part of the intracellular machinery for peroxide detoxification [21]. The number of copies of mitochondrial DNA per cell was investigated in parallel, since it has been suggested to increase with oxidative stress [22] and respiratory-function decline during aging [23].

## Materials and Methods

### 1. Cell culture

#### 1.1. Materials

Normal Human Dermal Fibroblasts (NHDF) from juvenile foreskin, fibroblast growth medium lacking ascorbic acid (FGM –AA) and fibroblast detach kit were purchased from PromoCell GmbH (Heidelberg, Germany). L-buthionine-[S, R]-sulphoximine (BSO) and L-glucose were from Sigma-Aldrich (Steinheim, Germany). IL1B was purchased from Peprotech (London, UK) and D-glucose from Peprotech (London, UK).

#### 1.2. Cell cultures

NHDFs were cultured in FGM –AA in a humidified incubator with an atmosphere of 5% CO_2_ at 37°C. The growth medium was especially made without ascorbic acid, in order to exclude the antioxidant effect of ascorbic acid on the pro-oxidant effect of the treatments applied subsequently in the present experiment. First passage cryopreserved NHDF from four genetically distinct donors were expanded for 13 days (up to passage 4). The four donors were 3, 14, 2 and 2 years old respectively. From each donor's cell culture at passage 4 five independent cultures were initiated and serially passaged for 90 days under five different treatments i.e. 20 independent cultures (four donors x five treatments). The five different treatments were: 1) FGM –AA only (control). 2) FGM –AA +10 μM BSO (pro-oxidant treatment). As shown in the study of Kurz et al. [20] a regular treatment with 10 μM BSO is adequate to induce intracellular oxidative stress but has no cytotoxic effect. 3) FGM –AA +25 mM D-glucose (high glucose treatment). We chose to apply the highest concentration of D-glucose among those used in the literature in order to ensure that the lack of an effect on ROS production would not be due to insufficient glucose concentration [12], [13], [14]. 4) FGM –AA +1 ng/ml IL1B (pro-inflammatory conditioning). The dose of IL1B was selected based on the data provided by the study of Busik et al. [12], [13], [14]. 5) FGM –AA +25 mM D-glucose +1 ng/ml IL1B (combination). In addition an extra culture from each donor was grown separately in FGM –AA containing 25 mM L-glucose in order to control for any osmotic effect caused to cells by the high glucose concentration in medium. The medium containing each treatment was changed every three days. In every passage, 600,000 cells were seeded into the new flask (175 cm^2^) i.e. the cell seeding density each time was ∼3,500 cells/cm^2^, which is within the recommended range of seeding density for NHDF. The number of population doubling (PD) was calculated using the formula PD  =  [*ln* (number of cells harvested) – *ln* (number of cells seeded)]/*ln*2 and the cumulative PD (CPD) by progressively adding the PD in each passage.

### 2. Telomere length measurement

At each passage a sample of the cells harvested from each of the 20 cultures (four donors x five treatments) was used for total DNA extraction with the PUREGENE DNA kit (Qiagen, West Sussex, UK). The extracted DNA samples were frozen at −20°C until measurement, without repeated freeze–thawing cycles. Mean telomere length was measured in these DNA samples using a validated quantitative PCR-based method as previously described [24]. We chose to measure mean telomere length in cell cultures generated from all the four available donors (four biological replicates) in order to acquire a large number of measurements for the statistical analysis of mean telomere length change over time. Briefly, the relative telomere length was calculated as the ratio of telomere repeats to single-copy gene (SCG) copies (T/S ratio). The SCG used was the genomic acidic ribosomal phosphoprotein PO (*36B4* or *RLP0*). For each sample the quantity of telomere repeats and the quantity of SCG copies were determined in comparison to a reference sample in a telomere and a SCG quantitative PCR, respectively. The specificity of all PCRs was determined by melting curve analysis and the relative concentration of the PCR products was calculated using the comparative quantification analysis (Rotor-Gene 6000 software, Corbett Research Ltd, Cambridge, UK). All PCRs were performed on the Rotor-Gene 6000 (Corbett Research Ltd, Cambridge UK) in duplicates. The inter-assay coefficient of variation in repeated measurements was 5.6%.

### 3. Mitochondrial DNA (mtDNA) copy number measurement

The DNA samples extracted as described above from each of the 20 cultures (four donors x five treatments) at each passage were also used for the estimation of the copy number of mtDNA per nucleus, which is representative of the mitochondria number per cell. Again, we chose to measure mtDNA copy number in cell cultures generated from all the four available donors (four biological replicates) in order to acquire a large number of measurements for the statistical analysis of mtDNA copy number change over time. For this purpose a quantitative PCR-based method was used [25]. The relative number of mtDNA copies per genomic DNA copies (M/G ratio) was calculated as the ratio of the mitochondrial subunit 1 (*MTND1*) gene copies to the genomic *RLP0* gene copies. These genes are single copy genes for the mtDNA and the genomic DNA respectively. The number of mtDNA copies and genomic DNA copies in each sample was determined in comparison to a reference sample in a *MTND1* and a *RLP0* quantitative PCR, respectively. All PCRs were performed on the Rotor-Gene 6000 (Corbett Research Ltd, Cambridge UK). The relative concentrations of PCR products were estimated using the comparative quantification analysis (Rotor-Gene 6000 software, Corbett Research Ltd, Cambridge, UK). The second derivative of the amplification curve was considered in order to identify the peak of the exponential amplification and determine the Take-Off of the reaction. The Take-Off was estimated by finding the first point to be 80% below the peak level. Based on the Take-Off point and the amplification, the software calculated the relative quantity of *MTND1* and *RLP0* copies in each sample compared to the reference sample. The same reference DNA was used in all runs to allow comparison of the results in different runs. The *MTND1* and the *RLP0* PCRs for each sample were performed in duplicate in the same run in order to increase accuracy. The specificity of all amplifications was determined by melting curve analysis.

The primers, used for the *MTND1* amplification, were: forward: 5′-TGGGTACAATGAGGAGTAGG-3′, reverse: 5′-GGAGTAATCCAGGTCGGT-3′ at a 215 nM concentration and for the *RLP0* these were: forward: 5′-CCCTAAAACCCGCCACATCT-3′, reverse: 5′-GAGCGATGGTGAGAGCTAAGGT-3′ at a 300/500 nM (forward/reverse) concentration. For both PCRs the cycling profile was 95°C incubation for 10 min, followed by 34 cycles of 95°C for 15 sec and 58°C for 60 sec and the final reaction consisted of 1xqPCR mix (2× SensiMix NoRef DNA kit, Quantace, London, UK), 30 ng of template and the respective primer concentrations at a 25 μl total volume.

A dilution series (1.25 ng/μl –80 ng/μl, two-fold dilution, seven points) was run after optimization for both the *MTND1* and *RLP0* PCRs. For both assays, linearity (R^2^>0.99) over this range of input DNA and 100% efficiency was observed (Figure S1 panels A and B).

In order to test the reproducibility of the method, 16 randomly chosen samples were run in duplicates on two consecutive days. There was a significant linearity between the measurements obtained on the two different days in linear regression analysis (R^2^ = 0.52, p = 0.002, Figure S1 panel C). Moreover, the reproducibility was also assessed with Spearman's non-parametric test of pair-wise correlation that looks at the ranking of each sample. The correlation of the mtDNA copy number ranking as measured on the two different days was significant (Spearman rho coefficient = 0.69, p = 0.003). The coefficient of inter-assay variation in repeated measurements was 7.4%.

### 4. Estimation of cell death

Annexin-V-FLUOS Staining Kit (Roche Diagnostics GmbH, Penzberg, Germany) was employed for the quantification of dying and dead cells. The percentage of dying and dead cells was evaluated in NHDF cultures under each of the five treatments (as described above) generated from two randomly selected donors after a period of seven days. The cells detached by trypsinisation were washed with PBS, collected by centrifugation, resuspended in Annexin-V-FLUOS (which binds to the phosphatidylserine exposed upon the outer leaflet of the cell membrane of dying cells) and/or propidium iodide (PI) (which binds to the exposed DNA of dead cells) labeling solution and incubated for 15 min at 15–25°C. The samples were then analysed on a FACSCalibur (Becton-Dickinson) flow cytometer using 488 nm excitation with a 530 nm filter for fluorescein detection and a 585 nm filter for PI detection. Electronic compensation of the instrument was performed before each measurement. Data were acquired and analysed with the Cellquest Pro software (Becton Dickinson Biosciences, Oxford, UK). A representative graph is shown in panel A of Figure S2. A second independent measurement in each of the ten cultures (two donors x five treatments) was performed in order to verify our observations.

### 5. Measurement of intracellular ROS content

The ROS detection reagent 2′,7′-dichlorodihydrofluorescein diacetate (H_2_DCF-DA) was purchased from Molecular Probes (Invitrogen, Oregon, USA). H_2_DCF-DA is widely used cell-permeate indicator for a large range of reactive oxygen species (e.g. hydrogen peroxide, peroxyl radical and peroxynitrite anion) [26]. The H_2_DCF-DA indicator was reconstituted in 100% ethanol shortly before performing the measurement. The intracellular ROS was evaluated in NHDF cultures under each of the five treatments (as described above) generated from two randomly selected donors after a period of seven days. The cells detached by trypsinisation were washed with PBS, collected by centrifugation, resuspended in PBS buffer containing H_2_DCF-DA at a 10 μM concentration and incubated for 10 min at 15–25°C. Then the buffer was removed and cells were returned in pre-warmed PBS. The 5-(and-6)-carboxy-2′,7′-dichlorodihydrofluorescein diacetate (carboxy-DCFDA) (from Molecular Probes, Invitrogen, Oregon, USA) was used as a positive control. The samples were then analysed on a flow cytometer using 488 nm excitation and a 530 nm filter for fluorescein detection. Data were acquired and analysed with the Cellquest Pro software (Becton Dickinson). Dying and dead cells, as detected with Annexin-V/PI staining, were excluded from the estimation of ROS content. A representative graph of the quantification of the ROS content in viable cells is shown in in panel B of Figure S2. A second independent measurement in each of the ten cultures (two donors × five treatments) was performed in order to verify our observations.

### 6. Gene expression assays

Total RNA was extracted using the RNeasy Mini kit (Qiagen, West Sussex, UK) from cells harvested from each of the 20 cultures (four donors × five treatments) at three time points; before any treatment was initiated, after six days and after 45 days of treatment. Cell cultures generated from all the four available donors were included in these assays in order to acquire an adequate number of biological replicates for the estimation of gene expression changes in three different time points. RNA quantity and purity was assessed using the nanodrop spectrophotometer ND-8000 (Labtech, East Sussex, UK). cDNA was synthesised using the Superscript III Reverse Transcriptase (Invitrogen, Paisley, UK) according to the manufacturer's protocol. Briefly, 100 ng of RNA were incubated with random primers and 10 mM dNTP for 5 min at 65°C. Dithiothreitol (DTT) and reverse transcriptase were then added and incubated for 1 h. The level of expression of the telomere reverse transcriptase (*TERT*) and the mitochondrial transcription factor A (*TFAM*) genes were estimated using TaqMan technology. The probes used were: *TERT* (Hs01082775_m1), *TFAM* (Hs00162669_m1) and for the housekeeping genes: ubiquitin C (*UBC*) (Hs00824723_m1), beta actin (*ACTB*) (Hs99999903_m1) and glyceraldehydes-3-phosphate dehydrogenase (*GAPDH*) (Hs99999905_m1) (Applied Biosystems, Cheshire, UK). The qPCR reactions were performed on the ABI prism 7900HT sequences detection system (Applied Biosystems, Cheshire, UK), in triplicate, and the results were analysed with the S.D.S.2.1 Applied Biosystems software for relative quantification. The BestKeeper software [27] was used to test the suitability of the three housekeeping genes. The relative expression ratio of target genes and the corresponding p values were estimated, after standardisation with the three house-keeping, using the REST software as previously described [28].

### 7. Statistical analysis

Statistical analysis was performed with SPSS statistical software (version 17.0 for Windows). Telomere length and mtDNA copy number were log-transformed (natural log) to a normal distribution. The differences in CPD, telomere length and mtDNA copy number among the cultures generated from the four different donors were tested with univariate analysis of variance using the pooled data and adjusting for treatment. The data from the four independent experiments, generated from the four donors, were analysed pooled after adjusting for donor as well as on a per donor basis. The association of the days of treatment with telomere length and mtDNA copy number as well as the association of CPD with telomere length and mtDNA copy number were evaluated using partial correlation coefficients controlling for donor. To assess the effect of each treatment on the telomere length change over time, we used a linear regression model with telomere length as the dependent variable and the days of treatment, the donors and the different treatments as the independent variables. The treatments were introduced in the model as dummy variables by leaving out of the model the dummy variable corresponding to the control. This was done in order to force the comparison of each treatment to the control, since this was our hypothesis. The same model with the CPD or the mtDNA as independent variables was used to test the effect of the treatments on the growth rate (i.e. the CPD change over time) or the change of the mtDNA copy number over time, respectively. In order to examine the effect on telomere length or the mtDNA copy number of each treatment compared to the control adjusting for the CPD, we used the respective linear regression models as described above, by replacing the days of treatment with the CPD in the list of independent variables. The p values from the comparison of each treatment with the control were obtained from the multivariable regression models described above. The percentages of change in telomere length and mtDNA per day or PD, as well as the PD per day presented, were obtained from separate analysis for each treatment regression model adjusted for donor. Regarding the comparisons between the percentages of dying and dead cells, as well as the intracellular ROS content of each treatment with the control, non parametric tests were used due to the small number of measurements. The Kruskal-Wallis test was used for comparisons among all treatments and the Mann-Whitney test for the comparison of each treatment with the control. The relevant data are presented as median with inter-quartile range (IQR) of the measurements from different donors in each of the two independent experiments performed. Statistical significance was taken as p<0.05.

## Results

### 1. General characteristics of cultures

Fibroblasts were serially passaged for approximately 90 days. The general morphology of fibroblasts in cultures was that of elongated spindle-shaped cells having a branched cytoplasm, which is a normal morphology for skin fibroblasts. This normal morphology was retained in all cultures for the time course of 90 days. Also, no osmotic effect was observed in cultures treated with L-glucose compared to the control cultures. Figure S3 shows pictures of the fibroblast cultures, after 62 days of treatment in each of the experimental conditions, taken with a “Zeiss Axioshop 2” microscope.

### 2. Growth rate

**Figure 1:**
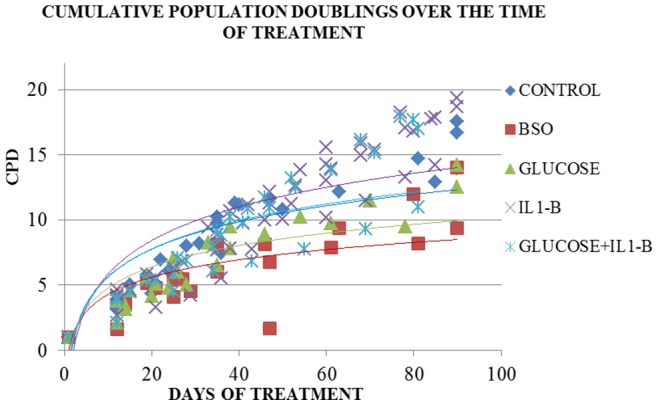
The cumulative population doublings (CPD) over the time of culture in each treatment (growth rate).

The cultures generated from the four different donors displayed significantly different growth rate, as reflected by the number of cumulative population doublings (CPD) (p = 0.04) occurring during the course of the experiment. The rate of growth was also significantly different among cultures with different treatments (p = 0.001), after adjusting for donor. A lower growth rate was observed in cultures treated with high glucose and BSO compared to the control [Glucose: 0.14 PD/day, p<0.001 and BSO: 0.11 PD/day, p = 0.006 vs. Control: 0.16 PD/day], whereas, cultures treated with IL1B displayed a trend towards a higher cell turnover compared to the control [IL1B: 0.19 PD/day, p = 0.093 vs. Control: 0.16 PD/day] (Table 1, Figure 1).

**Table 1:** Percentage changes in mean telomere length and mtDNA copies per nucleus over the time of culture (days) or cell divisions occurred during the experiment (CPD).

	Telomere Length				mtDNA				CPD	
	%/day (95%CI)	P	%/CPD (95%CI)	P	%/day (95%CI)	P	%/CPD (95%CI)	P	CPD/day (95%CI)	P
**CONTROL**	−0.6 (−0.8, −0.3)		−2.5 (−4.4, −0.7)		0.1 (−0.1, 0.4)		1.5 (0.0, 3.1)		0.16 (0.14, 0.18)	
**BSO**	−0.2 (−0.6, 0.2)	0,112	−0.9 (−4, 2.2)	0.843	0.2 (−0.2, 0.6)	**0.047**	2.3 (−0.8, 5.6)	**0.042**	0.11 (0.09, 0.13)	**0.006**
**GLUCOSE**	−0.4 (−0.7, 0.0)	0,685	−2.3 (−4.7 ,0.0)	0.341	0.1 (−0.4, 0.5)	0.436	0.1 (−2.9, 2.8)	0.442	0.14 (0.13, 0.16)	**<0.001**
**IL1B**	−0.8 (−1.1, −0.5)	**0,012**	−4.1 (−5.7, −2.4)	**0.067**	0.1 (−0.1, 0.3)	0.210	0.3 (−0.7, 1.4)	0.461	0.19 (0.18, 0.2)	**0.093**
**GLUCOSE +IL1B**	−0.8 (−1.2, −0.4)	0,156	−4.3 (−6.3, −2.4)	0.169	0.3 (0.0, 0.6)	0.136	1.2 (−0.4, 2.9)	0.230	0.18 (0.17, 2.0)	0.926

%: percentage change, CPD: cumulative population doublings, mtDNA: mitochondrial DNA, IL1B: interleukin 1B, BSO: buthionine sulphoximine.

Percentage changes (%) in telomere length or mtDNA with days or CPD are obtained from separate regression models for each treatment adjusted for donor.

CPD per days are also obtained from separate regression models for each treatment adjusted for donor.

P values for the percentage changes (%) over days or CPD are obtained from regression models including all treatments as dummy variables compared to the control, adjusting for donor.

Similar results were observed when growth curves were plotted separately and the effect of the treatments was compared on a donor per donor basis, as shown in panel A of Figure S4. More specifically, the growth rate under IL1B treatment was significantly higher compared to control in two out of four donors (donor 1: p = 0.03 and donor 4: p = 0.09). BSO treatment caused a significantly lower growth rate compared to the control in three out of four donors (donor 1: p<0.001, donor 2: p<0.001 and donor 3 p = 0.03) and high glucose in one out of four donors (donor 1: p = 0.05).

Culture growth reached a plateau phase in all treatments and donors the latest between the 80th and the 90th day of culture (Figure 1A). However, as shown in panel A of Figure S4, the proliferative capacity was exhausted earlier in some cultures, such as the donor 3 culture under glucose treatment and the donor 4 culture under BSO treatment.

The growth rate observed over the time course of 90 days (control cultures: 0.16 PD/day) is relatively low. This can be attributed to the long term culture, since the growth rate got progressively lower with the serial passages and was minimized towards the end of the experiment. We observed that the rate of growth was higher at the beginning of the experiment and declined dramatically after ∼30 days of culture. It is important to note here that the cell seeding density was consistent in each passage (∼3,500 cells/cm^2^) and that the cultures were always passaged when a ∼90% confluence was observed. Therefore, the decline in growth rate over the time course of the experiment is probably due to exhaustion of the proliferative capacity of the cells.

### 3. Telomere length

**Figure 2:**
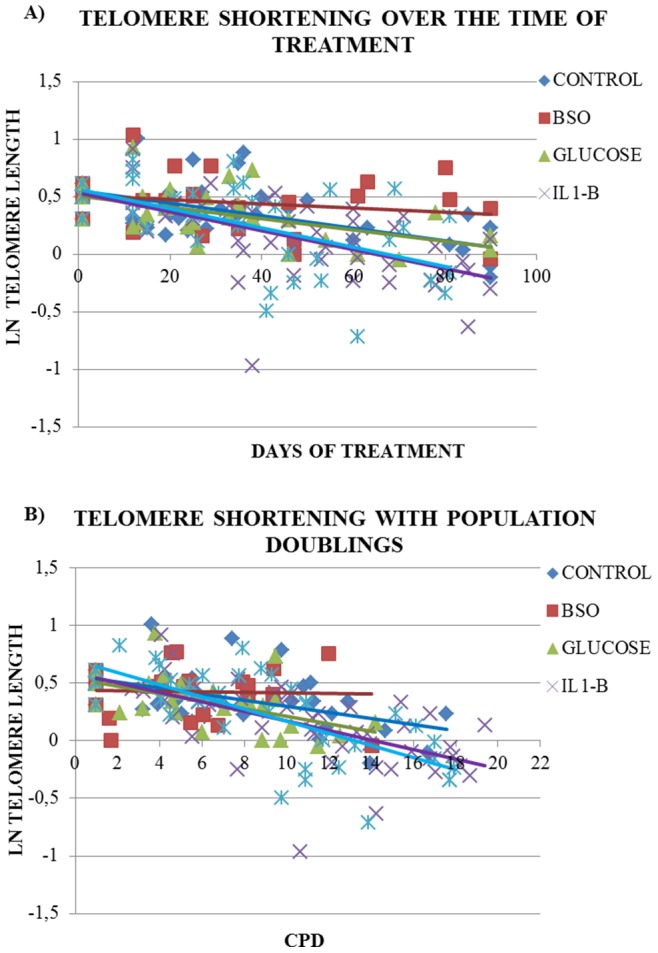
The shortening of mean telomere length during each treatment. Panel A shows the shortening of mean telomere length over the time of culture in days and Panel B the shortening of mean telomere length over the number of cell divisions occurred during the experiment, as reflected by the cumulative population doublings (CPD).

As expected, mean telomere length displayed a strong negative correlation with the number of days of culture (r = −0.65, p<0.001) and the CPD (r = −0.59, p<0.001) when the data from all treatments were pooled. Mean telomere length differed significantly between the cell cultures from the four different donors (p = 0.008). After adjusting for donor, the rate of telomere shortening over the time of treatment was significantly higher in cells treated with IL1B compared to the control [IL1B: −0.8%/day (95%CI: −1.1, −0.5) vs. Control: −0.6%/day (95%CI: −0.8, −0.3), p = 0.012] (Table 1, Figure 2A). No significant differences were found with the other treatments compared to control.

The effect of the treatments on telomere length was also compared on a donor per donor basis, and the observed results were similar to the pooled data analysis. A significantly higher rate of telomere shortening was observed in cultures treated with IL1B in two out of the four donors (donor 1: p = 0.05, donor 3: p = 0.01). Nevertheless, when the curves of telomere shortening are plotted separately for each donor, the cultures treated with IL1B displayed an accelerated telomere shortening in all donors (panel B of Figure S4). Also the combination of treatment with high glucose and IL1B caused a higher rate of telomere shortening compared to the control in donors 1, 3 and 4, which reached the level of significance only in donor 1 (p = 0.06).

In order to examine whether the effect of each treatment on telomere shortening was caused by their effect on cell turnover, we tested the differences in the decline of telomere length with each treatment, adjusting for CPD. The effect of IL1B on telomere shortening was attenuated only to some extent and retained its trend [IL1B: −4.1%/PD (95%CI: −5.7, −2.4) vs. Control: −2.5%/PD (95%CI: −4.4, −0.7), p = 0.067] (Figure 2B and Table 1). The respective regression models in each donor separately also showed that the adjustment for CPD did not change the effect of IL1B on telomere shortening. This effect of IL1B compared to the control remained significant in donors 1 and 3 (p = 0.03 and p = 0.05, respectively) as before the adjustment. In addition, the effect of the combination of high glucose and IL1B on telomere shortening maintained its trend in donor 3 after the adjustment for CPD (p = 0.06).

### 4. MtDNA copy number per nucleus

**Figure 3:**
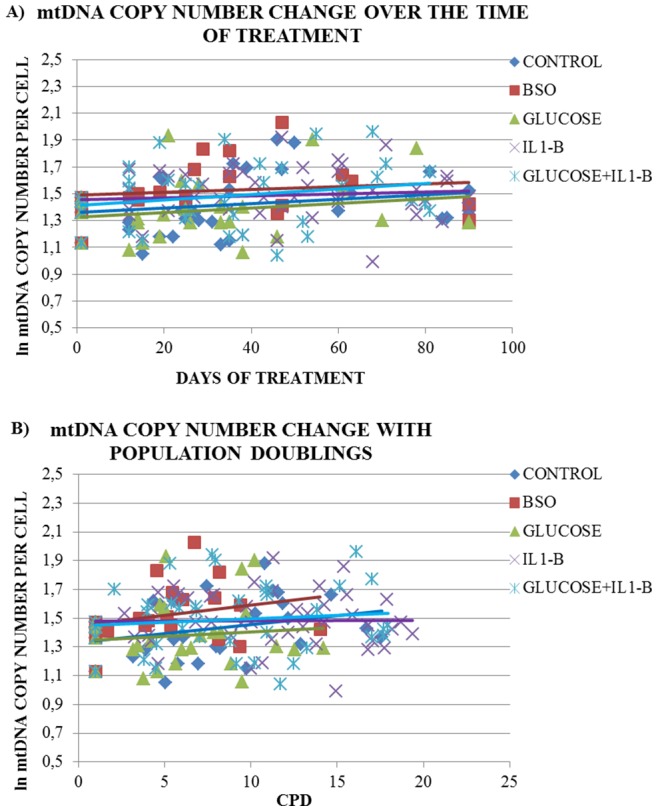
The change in the number of mitochondria per cell, as reflected by the copy number of mtDNA per nucleus, during each treatment. Panel A shows the change in the number of mitochondria per cell over the time of culture in days and Panel B the change in the number of mitochondria per cell over the number of cell divisions occurred during the experiment [i.e. cumulative population doublings (CPD)].

In all treatments, a statistically significant increase in the number of mtDNA copies per nucleus over time was observed (r = 0.19, p = 0.019). A modest increase in the number of mtDNA copies per nucleus was also observed with the number of cell divisions (r = 0.16, p = 0.056). The cultures from the four donors displayed significant differences in the number of mtDNA copies per nucleus (p = 0.041). After adjusting for donor, treatment with BSO resulted in a greater increase in the number of mtDNA copies per nucleus over the time of culture compared to the control [BSO: 0.2%/day (95%CI: −0.2, 0.6) vs. Control: 0.1%/day (95%CI: −0.1, 0.4), p = 0.047] (Table 1, Figure 3A). The effect of the treatments on telomere length was also compared on a donor per donor basis. Treatment with BSO resulted in a significantly higher increase in the number of mtDNA copies per nucleus over the time of culture compared to the control in donor 2 (p = 0.02). This effect was only observed in cultures generated from donor 2, whereas the regression models testing the change in mtDNA copies over the time of culture in the three other donors were not significant. When the curves of mtDNA copies change over time are plotted separately for each donor, there is no specific pattern of the treatments effect in all four donors (panel C of Figure S4).

The effect of BSO was not altered when adjusting for CPD in both the pooled data analysis [BSO: 2.3%/PD (95%CI: −0.8, 5.6) vs. Control: 1.5%/PD (95%CI:0.0, 3.1), p = 0.042] (Table 1, Figure 3B) and the the donor per donor analysis (Donor 2: BSO vs. control, p = 0.01). No other treatment had a statistically significant effect on the number of mtDNA copies per nucleus compared to the control.

### 5. Cell death

**Figure 4:**
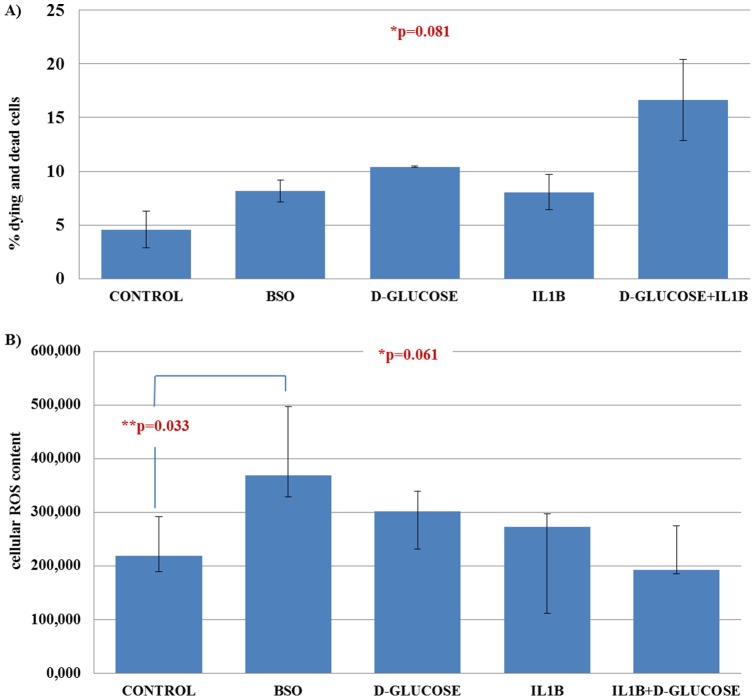
The cell death and intracellular ROS content as measured by flow cytometry after 7 days of culture in each treatment. The percentage of dying and dead cells is shown in panel A and the intracellular ROS content of viable cells in panel B. *Figure 4* *footnote*: Due to the small number of measurements, normal distribution cannot be inferred. Thus, the graph represents median values, with inter-quartile range as error bars. *P value is obtained from Kruskal-Wallis test. **P value is obtained from Mann-Whitney test. Mann-Whitney tests between the percentages of dying and dead cells in each of the treatments compared with the control were non-significant (p = 0.121).

In both the independent measurements of cell death performed we observed a higher percentage of dying and dead cells was observed in each of the five treatments compared to the control after seven days of treatment. The results from one of the independent measurements are presented in figure 4A. The difference between the control and each treatment was not statistically significant (p = 0.121). However, overall there was a trend in the percentages of dying and dead cells (p = 0.081), which appears to be driven by the effect of the combination of high glucose and IL1B. The pattern was similar in the other independent experiment.

### 6. Intracellular ROS production

Figure 4B shows the results from one of the two independent measurements performed. BSO treatment for seven days induced in fibroblasts a significantly higher production of ROS compared to the control (p = 0.033). The intracellular ROS content was similar in cells treated with all the other treatments and the control (p>0.2), with a similar pattern observed in a second independent measurement.

### 7. Gene expression

#### TERT

Human skin fibroblasts do not normally express telomerase [19]. In order, to examine whether telomerase expression was induced by the different treatments, expression levels of *TERT*, the gene encoding the catalytic part of telomerase, were measured in each culture by RT-PCR. No quantifiable expression of *TERT* was detected before applying any treatment, nor at the first days of treatment (six days of treatment), or towards the end of the experiment (45 days of treatment), in any of the five treatments.

#### TFAM

In order to examine whether the increase in the mtDNA copies in the cells treated with BSO reflected an enhancement of mitochondria proliferation, we examined the expression of *TFAM*, the gene encoding the transcription factor responsible for mitochondria proliferation. *TFAM* mRNA levels from cells before any treatment were used as control, in order to calculate the relative change in expression of *TFAM* after six and 45 days of treatment. The relative expression ratio of *TFAM* fell in the high glucose and IL1B treatments alone and in combination, but overall these differences were not statistically significant (Figure S5).

## Discussion

In the last few years an increasing number of epidemiological studies have been published on the association of telomere length with CVD and T2D [5]. However, whether chronic inflammation, which is implicated in the pathogenesis of both CVD [17] and diabetes [18], causes accelerated telomere shortening during *in vitro* ageing has not been investigated so far. The present study is the first, to our knowledge, to provide evidence suggesting that pro-inflammatory conditioning with IL1B exacerbates the shortening of telomeres in an *in vitro* model of human cellular aging. This effect of IL1B on telomeres was only in part driven by higher cell turnover. In the present model, IL1B induced a moderate increase in cell turnover, and its effect on telomere shortening was only in part attenuated after adjustment for the number of cell divisions in the cultures. This is further supported by the fact that between the two donors' cultures, where the effect of IL1B on the cells' telomere shortening was more pronounced, only in one donor's cultures IL1B treatment led to an increased cell turnover. Thus, it can be speculated that pro-inflammatory conditioning with IL1B might exacerbate the shortening of telomeres also through a still unknown pathway. The trigger of this proposed pathway may be a specific response to IL1B, or part of a general inflammatory response, such as is known to occur in early atherosclerosis and diabetes. Possibly, IL1B deregulates the expression or the interplay of proteins essential for the protective structure of telomeres, the “shelterin” complex, such as TRF2 [29]. Further experiments are needed to explore the mechanism by which pro-inflammatory conditioning exacerbates the shortening of telomeres employing different inflammatory factors, or testing the reversibility of this phenomenon using anti-inflammatory agents such as aspirin and statins.

The effect of pro-oxidant treatment with BSO was also examined in the present experiment. BSO inhibits glutathione synthesis, a process essential for the intracellular detoxification of peroxides [21]. As expected, the cultures of fibroblasts treated with BSO exhibited a higher intracellular ROS production compared to control cultures; however, the high ROS levels in these cultures did not result in greater telomere shortening. ROS are known to cause single DNA strand breaks in G-rich overhangs of telomeres, which are prone to oxidative damage and which are considered to lead to greater telomere shortening in each cell division [11]. Such telomeric loss does not seem to have occurred here. Instead, the ROS-induced single strand breaks appear to have led to a stress-associated, although telomere independent, arrest of the cell cycle [9]. This phenomenon has been described as “stasis”, where the cells are arrested at G1 phase and do not display genomic instability or critically short telomeres [30]. The occurrence of the phenomenon of “stasis” is evident in the cultures generated from donor 2, where the increase in mtDNA copies was accompanied by a pronounced lowering of the growth rate in cultures treated with BSO compared to the control. In order to verify whether this increase was due to induced proliferation of the mitochondria, in an effort to compensate for the ROS increase [23], we examined the expression of *TFAM*. *TFAM* is the transcription factor regulating the mitochondria proliferation, and its expression was not found to change significantly in BSO treated cultures compared to control cells. Thus, the observed increase in mtDNA content probably indicates an oxidative stress-induced cell cycle arrest at G1 phase rather than mitochondrial proliferation, as has been also described by Lee and colleagues [22]. The present findings are also supported by the study of Ksiazek et al. [31], which showed that high glucose–induced increase in ROS production was accompanied by double-stranded DNA breaks mainly localised to non-telomeric regions of the genome. This provides a rational for the lack of effect on telomere length and the cell cycle arrest in cultures treated with BSO, which could have been caused by double stranded DNA breaks in non-telomeric regions.

In the present experiment high glucose concentration, alone, did not result in faster telomere shortening. This is not surprising given that glucose did not seem to have a significant effect on ROS levels in our experiment. Our hypothesis was that high glucose, as a substrate for the mitochondrial oxidative phosphorylation, would hasten the input of reducing equivalents into the electron transport chain and would thus increase the ROS production; apparently this did not occur in our experimental model. The current literature provides conflicting evidence regarding this hypothesis. There are studies showing that hyperglycaemia elicits an increase in intracellular ROS production which is a trigger for pathways responsible for hyperglycaemia-induced cell damage [12], [13], [14]. However, other studies have provided data arguing that high glucose induces an increase in ROS generation [15], [16]. The experiments of Busik et al. [16] demonstrated in endothelial cells that glucose consumption, and thus the generation of ROS, increases only when high glucose concentration is combined with pro-inflammatory conditioning with IL1B. However, in the present experiments with fibroblasts, the combination of high glucose and IL1B treatment did not result in higher intracellular ROS content. It is possible that the sensitivity to ROS generation, or the stimulus for higher consumption of nutrients, depends on the type of cell. In addition, although the main source of intracellular ROS in most cell types is the mitochondria [22], the actual substrate causing greater ROS generation during its catabolism in the mitochondria, might vary. For example, fatty acids like palmitate have been shown to induce ROS generation in a variety of cells [32], thus experiments testing its effect on telomeres would be valuable.

The effect of the combination of high glucose and IL1B on telomere shortening was similar to the effect of IL1B treatment alone. High glucose did not result in higher ROS generation even in combination with IL1B, which could have had further aggravated telomere attrition. However, high glucose led to a lower cell turnover which might have caused a greater variation of telomere lengths in cultures treated with the combination. This variation is probably responsible for the decline in telomere length not being significantly different, when compared to the control. The lower growth rate observed in cultures treated with high glucose indicates that a telomere-independent cell cycle arrest must have occurred [30].

Limitations of our study need to be considered. We estimated the decline in mean telomere length in each culture, but any elongation in specific chromosomes or in telomeres of subpopulations of cells would have diluted the observed effect on the rate of shortening. Human skin fibroblasts are considered a model of replicative senescence since they do not normally express telomerase [19]; thus the telomerase-mediated elongation of telomeres was not expected. In order to verify this in our experiments, the expression of *TERT* was examined, and was undetectable both before and during the treatment in all cultures. Nonetheless, the possibility of alternative lengthening of telomeres by recombination (ALT), which was shown to occur in endothelial cells treated with BSO in the study of Kruz et al. [20], cannot be excluded. However, Kruz et al [20] reported that ALT occurred only at late passages, after ∼20 CPD, while in the present experiment the cultures treated with BSO were not grown beyond the 14 CPD, and in general, no cultures were kept beyond the 19 CPD. A possibility which cannot be excluded is that the high ROS levels induced by BSO might have caused single DNA strand breaks or critically short length on single telomeres in a subpopulation of cells in the culture or in specific chromosome arms. Nevertheless, the present study shows at least, that high oxidative stress does not cause systematic and gradual telomere shortening, in contrast to high inflammatory status.

## Conclusions

In conclusion, the novel aspect of the present study is showing that chronic inflammation, which characterizes cardiovascular disease and diabetes before and after their onset, may directly cause the shortening of telomeres, and thus result in premature senescence contributing to disease development and/or progression. This observation is of particular importance also in the context of other clinical conditions exhibiting a chromic pro-inflammatory state, such as autoimmune diseases. Worthy of remark is that short telomeres have been observed in patients with T1D [33], [34], rheumatoid arthritis [35] and systemic lupus erythematosus [36]. Understanding how telomere dynamics may link chronic inflammation to accelerated tissue ageing and consequent premature disease, will help us improve the means to prevention and treatment. Nevertheless, further studies are needed in order to improve our understanding on the mechanisms determining the shortening of telomeres and the consequent senescence in age-related diseases.

## Supporting Information

Figure S1 **Data supporting the method of Mitochondrial DNA (mtDNA) copy number measurement.** **A**) Mitochondrial single copy gene (*MTND1*) PCR standard curve. **B**) Genomic single copy gene (*RLP0*) PCR standard curve. **C**) Linear regression between measurements of mtDNA copy number measurement with the quantitative PCR in 16 samples acquired on two consecutive days (R^2^ = 0.52, p = 0.002).
(PDF)

Figure S2 **Representative graphs from flow cytometry measurements.** **A**) A representative graph of Annexin V/PI positive cells detection. **B**) A representative graph of the quantification of ROS content in viable cells.
(PDF)

Figure S3 **Microscope pictures of cultures, after 62 days of treatment in each of the experimental conditions.**
(PDF)

Figure S4 **The cumulative population doublings (CPD) [panel A], the shortening of mean telomere length [panel B] and the copy number of mtDNA per cell [panel C] over the time of culture in each treatment presented for each of the donors separately.**
(PDF)

Figure S5 **The change in** ***TFAM*** **expression levels after 45 days of treatment in each of** **the five conditions.**
(PDF)
