# Supplementary material for: The Effect of Pro-Inflammatory Conditioning and/or High Glucose on Telomere Shortening of Aging Fibroblasts
Source: PLoS One. 2013 Sep 23;8(9):e73756. doi: 10.1371/journal.pone.0073756 (PMC3781104; doi:10.1371/journal.pone.0073756)
Supplement: Figure S1 — Data supporting the method of Mitochondrial DNA (mtDNA) copy number measurement. A) Mitochondrial single copy gene (MTND1) PCR standard curve. B) Genomic single copy gene (RLP0) PCR standard curve. C) Linear regression between measurements of mtDNA copy number measurement with the quantitative PCR in 16 samples acquired on two consecutive days (R2 = 0.52, p = 0.002). (PDF) [file pone.0073756.s001.pdf]

Supporting Figure S1. Data supporting the method of Mitochondrial DNA (mtDNA) copy number measurement.

A) Mitochondrial single copy gene (*MTND1*) PCR standard curve. **B)** Genomic single copy gene (*RLP0*) PCR standard curve. **C)** Linear regression between measurements of mtDNA copy number measurement with the quantitative PCR in 16 samples acquired on two consecutive days (R^2=0.52, p=0.002).

A)

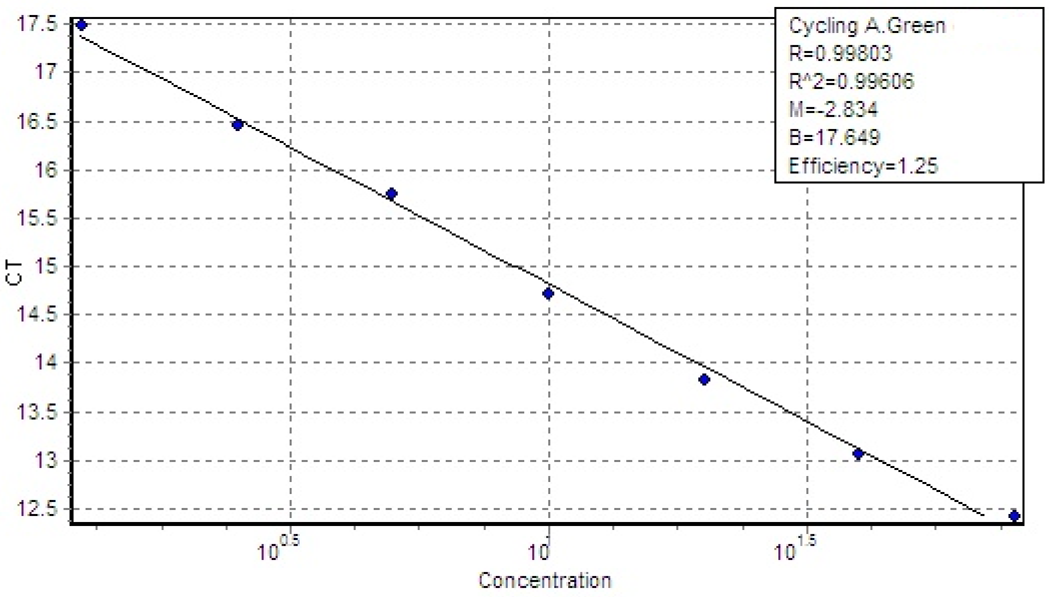

B)

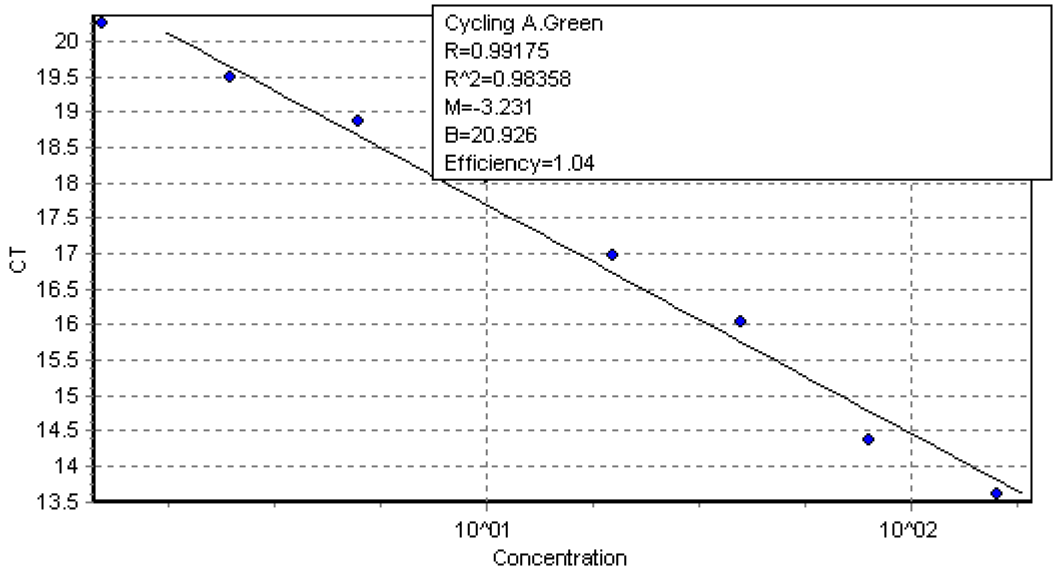

C)

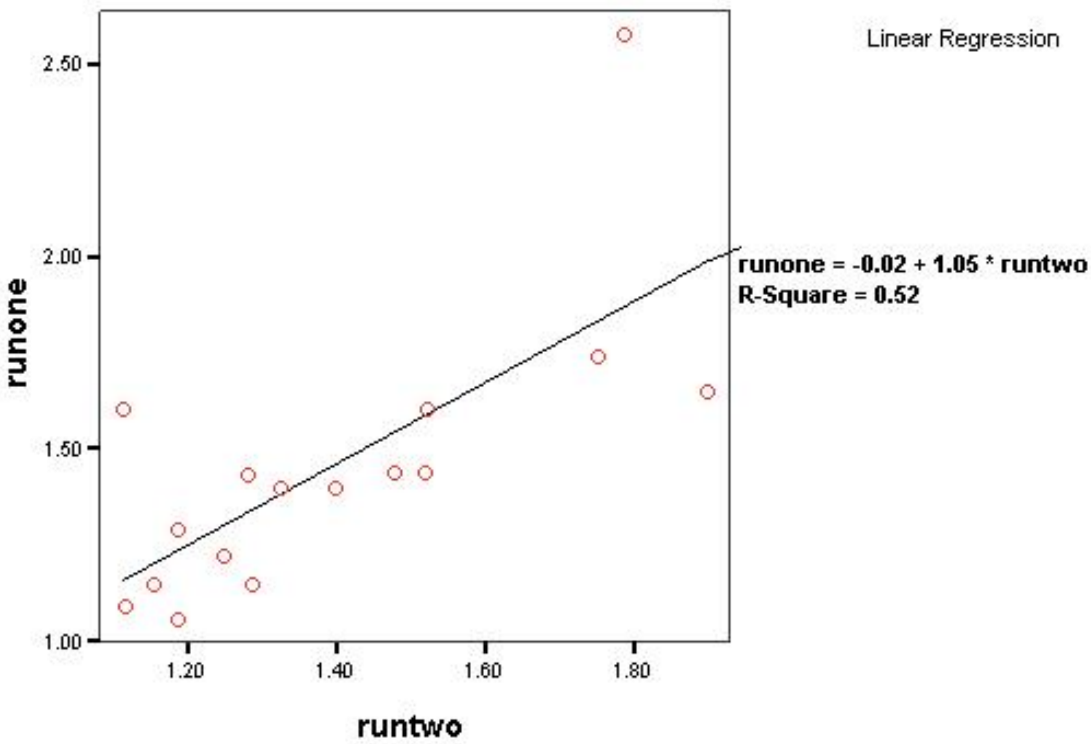
Linear Regression
runone = -0.02 + 1.05 * runtwo
R-Square = 0.52
2.50
2.00
1.50
1.00
runone
1.20
1.40
1.60
1.80
runtwo
