# Supplementary material for: The Effect of Pro-Inflammatory Conditioning and/or High Glucose on Telomere Shortening of Aging Fibroblasts
Source: PLoS One. 2013 Sep 23;8(9):e73756. doi: 10.1371/journal.pone.0073756 (PMC3781104; doi:10.1371/journal.pone.0073756)
Supplement: Figure S2 — Representative graphs from flow cytometry measurements. A) A representative graph of Annexin V/PI positive cells detection. B) A representative graph of the quantification of ROS content in viable cells. (PDF) [file pone.0073756.s002.pdf]

Supporting Figure S2. Representative graphs from flow cytometry measurements.

A) A representative graph of Annexin V / PI positive cells detection. **B)** A representative graph of the quantification of ROS content in viable cells.

A)

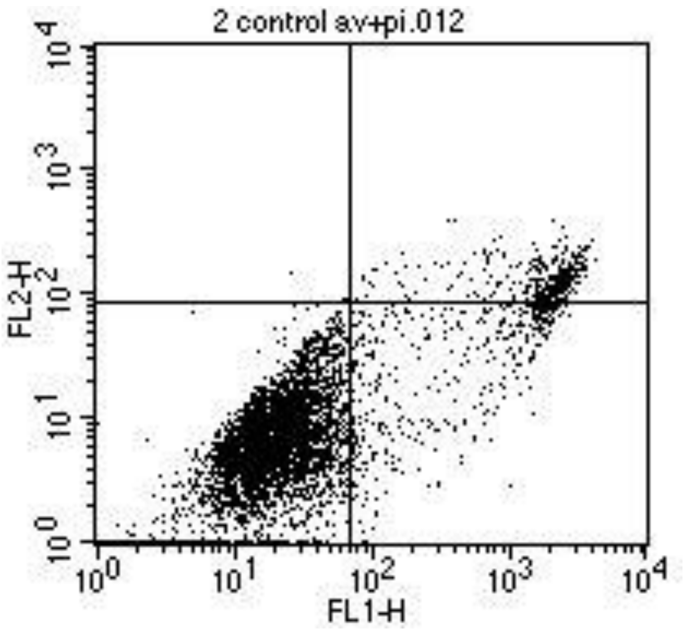

FL1 channel detects cells stained with annexin-V-FLUOS and FL2 detects cells stained with propidium iodide. Thus, the dying cells are presented on the lower right side of the cross, the dead cells on the upper right side and the upper left side. The viable cells are presented on lower left side of the cross.

B)

(i)

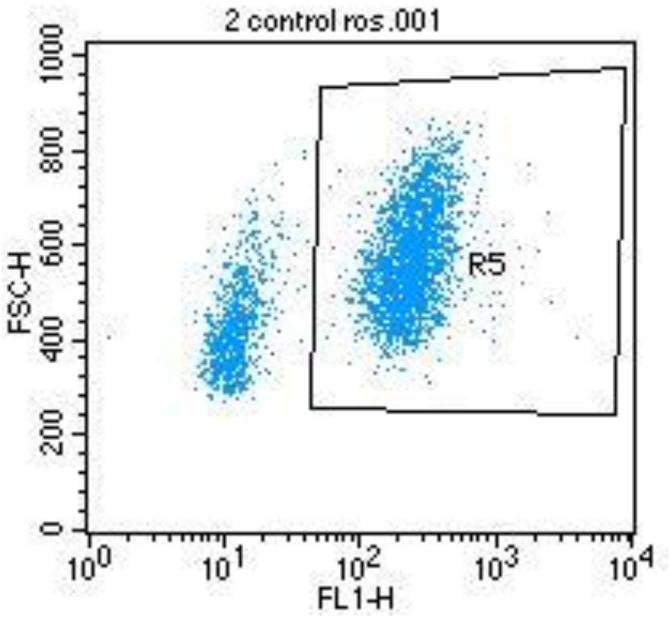

(ii)

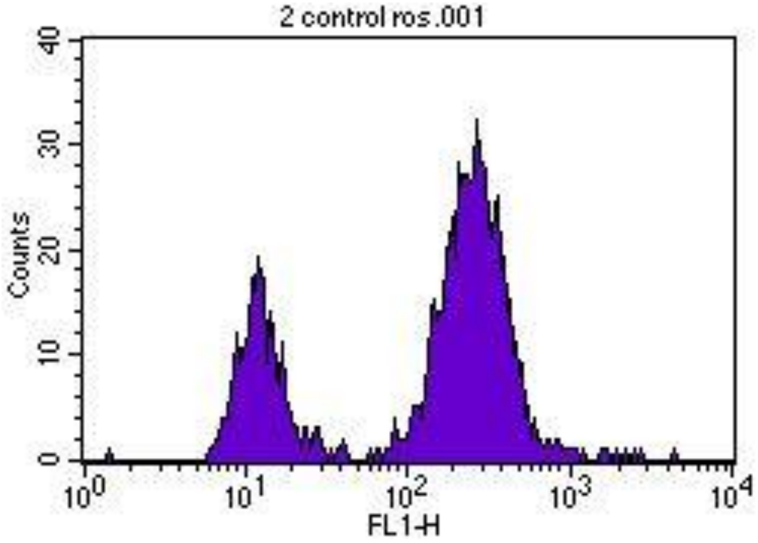

The H_2DCFDA dye is oxidised by the intracellular reactive oxygen species (ROS) and emits fluorescence detectable by FL1. Thus, the ROS content is estimated by the FL1 fluorescence intensity [as calculated in histogram (ii) by the distance between the peaks of the slopes of cells with and without the dye] multiplied by the number of viable cells emitting this fluorescence [as calculated in graph (i) by the number of cells shifting on the FL1 axis].
