# Supplementary material for: The Effect of Pro-Inflammatory Conditioning and/or High Glucose on Telomere Shortening of Aging Fibroblasts
Source: PLoS One. 2013 Sep 23;8(9):e73756. doi: 10.1371/journal.pone.0073756 (PMC3781104; doi:10.1371/journal.pone.0073756)
Supplement: Figure S3 — Microscope pictures of cultures, after 62 days of treatment in each of the experimental conditions. (PDF) [file pone.0073756.s003.pdf]

Supporting Figure S3. Microscope pictures of cultures, after 62 days of treatment in each of the experimental conditions.

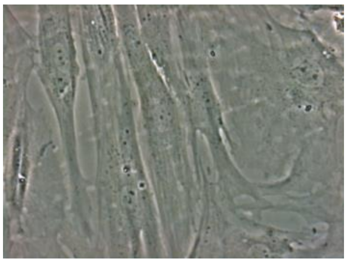

control

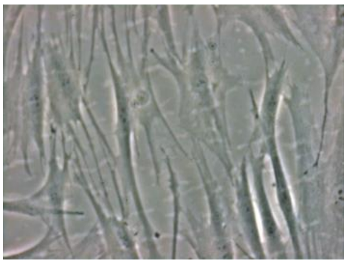

L-glucose

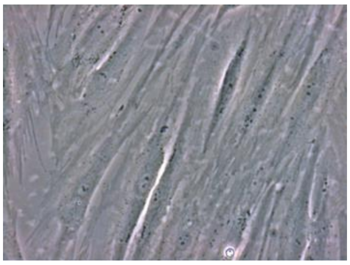

BSO

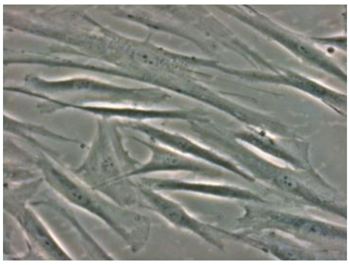

IL1B+ D-glucose

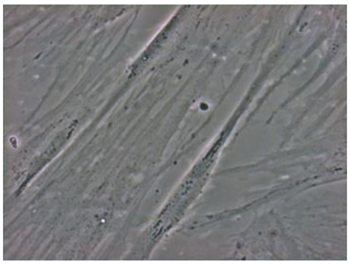

D-glucose

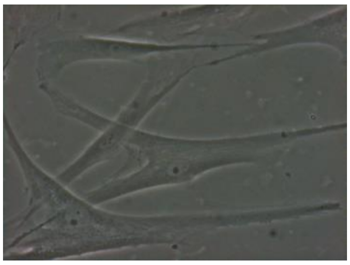

IL1B
