# Supplementary material for: The Effect of Pro-Inflammatory Conditioning and/or High Glucose on Telomere Shortening of Aging Fibroblasts
Source: PLoS One. 2013 Sep 23;8(9):e73756. doi: 10.1371/journal.pone.0073756 (PMC3781104; doi:10.1371/journal.pone.0073756)
Supplement: Figure S4 — The cumulative population doublings (CPD) [panel A], the shortening of mean telomere length [panel B] and the copy number of mtDNA per cell [panel C] over the time of culture in each treatment presented for each of the donors separately. (PDF) [file pone.0073756.s004.pdf]

Supporting Figure S4. **The cumulative population doublings (CPD) [panel A], the shortening of mean telomere length [panel B] and the copy number of mtDNA per cell [panel C] over the time of culture in each treatment presented for each of the donors separately.**

A)

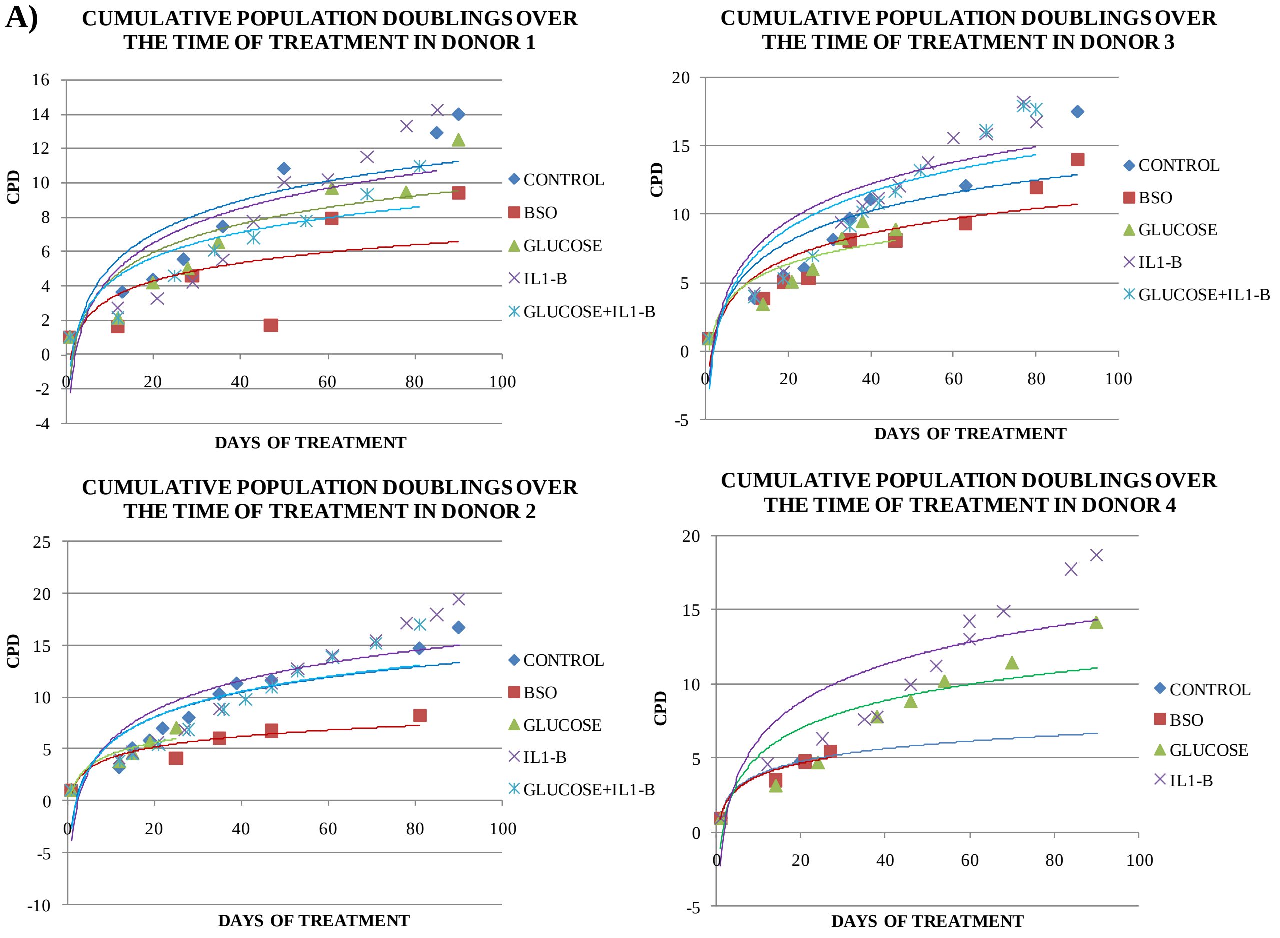

B)

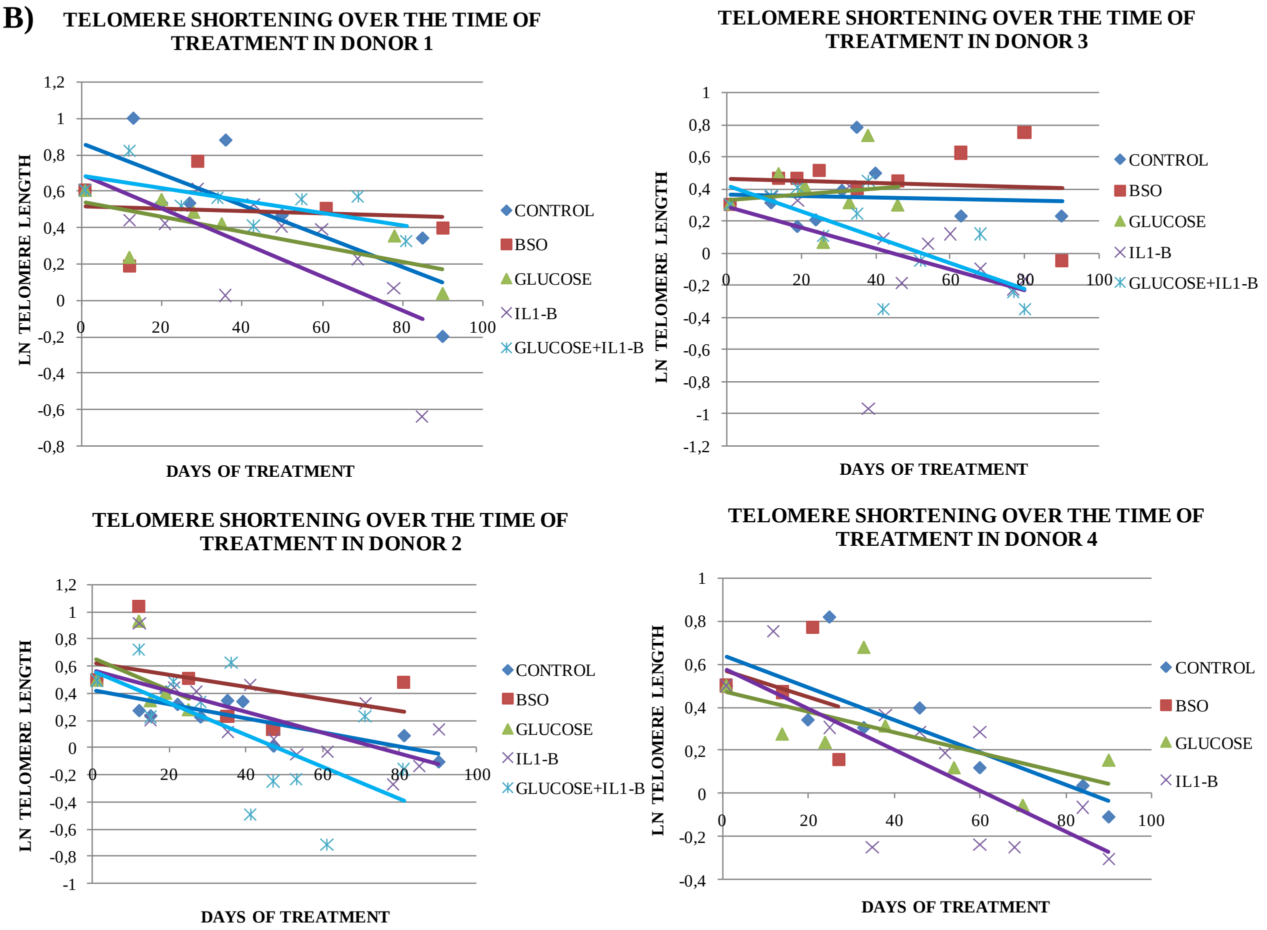
TELOMERE SHORTENING OVER THE TIME OF TREATMENT IN DONOR 1
LN TELOMERE LENGTH
DAYS OF TREATMENT
CONTROL
BSO
GLUCOSE
IL1-B
GLUCOSE+IL1-B
TELOMERE SHORTENING OVER THE TIME OF TREATMENT IN DONOR 3
LN TELOMERE LENGTH
DAYS OF TREATMENT
CONTROL
BSO
GLUCOSE
IL1-B
GLUCOSE+IL1-B
TELOMERE SHORTENING OVER THE TIME OF TREATMENT IN DONOR 2
LN TELOMERE LENGTH
DAYS OF TREATMENT
CONTROL
BSO
GLUCOSE
IL1-B
GLUCOSE+IL1-B
TELOMERE SHORTENING OVER THE TIME OF TREATMENT IN DONOR 4
LN TELOMERE LENGTH
DAYS OF TREATMENT
CONTROL
BSO
GLUCOSE
IL1-B

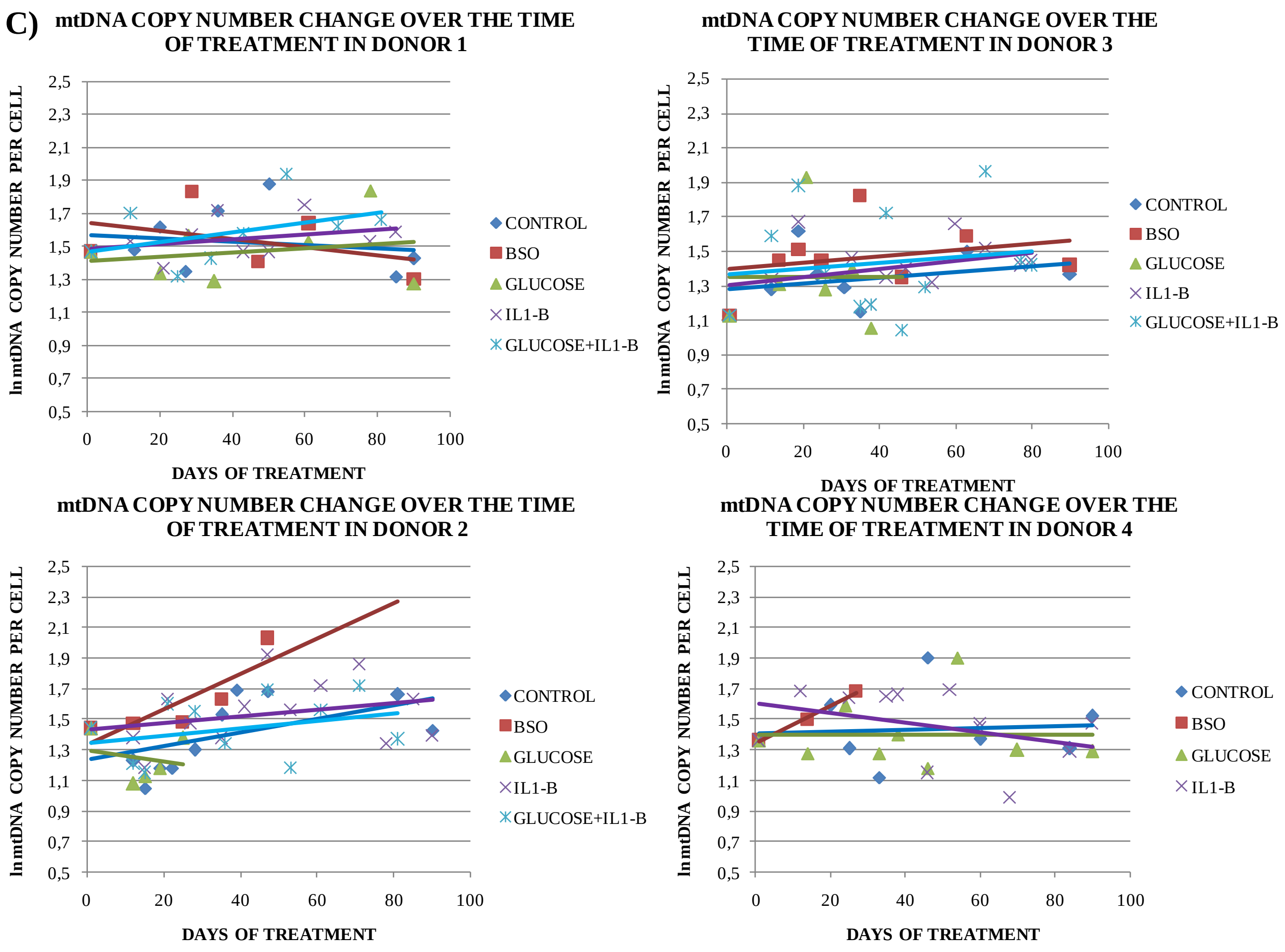

One of the cultures generated from donor 4, which was treated with the combination of high glucose and IL1B, was contaminated at day 25 of the culture period, thus the measurements from this culture were discarded from all analyses.
