# Supplementary material for: The Effect of Pro-Inflammatory Conditioning and/or High Glucose on Telomere Shortening of Aging Fibroblasts
Source: PLoS One. 2013 Sep 23;8(9):e73756. doi: 10.1371/journal.pone.0073756 (PMC3781104; doi:10.1371/journal.pone.0073756)
Supplement: Figure S5 — The change in TFAM expression levels after 45 days of treatment in each of the five conditions. (PDF) [file pone.0073756.s005.pdf]

Supporting Figure S5. The change in *TFAM* expression levels after 45 days of treatment in each of the five conditions.

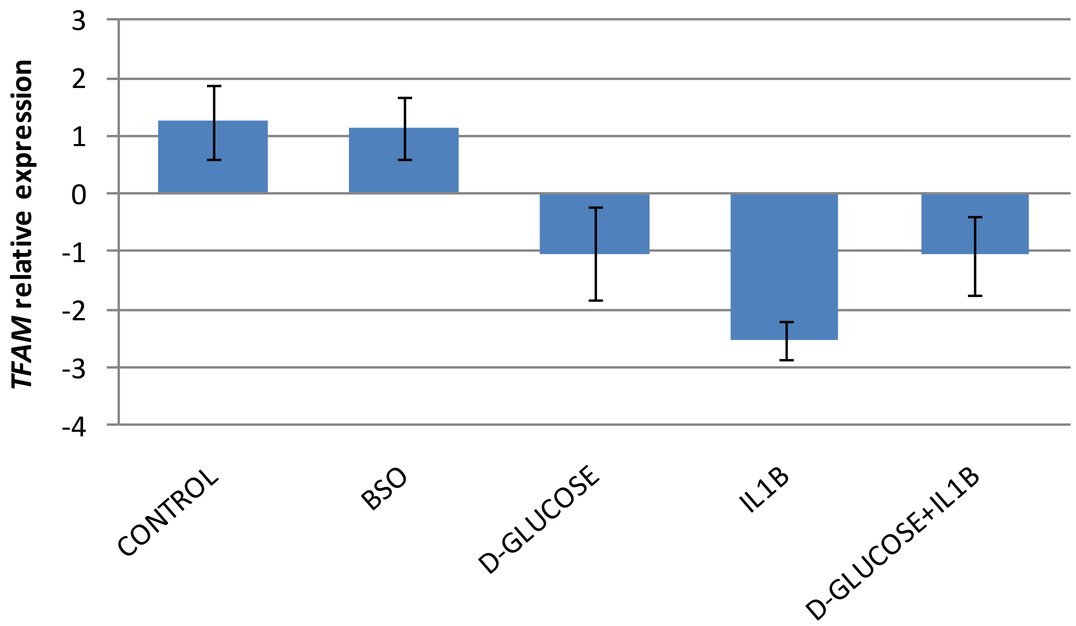

Expression ratio was calculated using the REST software.

The change in *TFAM* expression with each of the five treatments was calculated relatively to the levels of *TFAM* expression in each donor before the beginning of treatment.

There were no significant changes in *TFAM* expression with each of the five treatments.
